# Oxytocin receptor ligand binding in embryonic tissue and postnatal brain development of the C57BL/6J mouse

**DOI:** 10.3389/fnbeh.2013.00195

**Published:** 2013-12-11

**Authors:** Elizabeth A. D. Hammock, Pat Levitt

**Affiliations:** ^1^Vanderbilt Kennedy Center and Department of Pediatrics, Vanderbilt University School of Medicine, Vanderbilt UniversityNashville TN, USA; ^2^Department of Pediatrics, Children's Hospital Los Angeles and Keck School of Medicine of the University of Southern CaliforniaLos Angeles, CA, USA

**Keywords:** oxytocin, adrenal gland, kidney, oronasal cavity, autism, autoradiography, experience-dependent plasticity, neocortex

## Abstract

Oxytocin (OXT) has drawn increasing attention as a developmentally relevant neuropeptide given its role in the brain regulation of social behavior. It has been suggested that OXT plays an important role in the infant brain during caregiver attachment in nurturing familial contexts, but there is incomplete experimental evidence. Mouse models of OXT system genes have been particularly informative for the role of the OXT system in social behavior, however, the developing brain areas that could respond to ligand activation of the OXT receptor (OXTR) have yet to be identified in this species. Here we report new data revealing dynamic ligand-binding distribution of OXTR in the developing mouse brain. Using male and female C57BL/6J mice at postnatal days (P) 0, 7, 14, 21, 35, and 60 we quantified OXTR ligand binding in several brain areas which changed across development. Further, we describe OXTR ligand binding in select tissues of the near-term whole embryo at E18.5. Together, these data aid in the interpretation of findings in mouse models of the OXT system and generate new testable hypotheses for developmental roles for OXT in mammalian systems. We discuss our findings in the context of developmental disorders (including autism), attachment biology, and infant physiological regulation.

**Summary**: Quantitative mapping of selective OXTR ligand binding during postnatal development in the mouse reveals an unexpected, transient expression in layers II/III throughout the mouse neocortex. OXTR are also identified in several tissues in the whole late embryo, including the adrenal glands, brown adipose tissue, and the oronasal cavity.

## Introduction

There is substantial experimental evidence in humans and pre-clinical models that oxytocin (OXT) plays a role in adult behavior. In humans, whereas data from genetic association studies are difficult to replicate (Bakermans-Kranenburg and van Ijzendoorn, 2013), results from studies with intranasal treatment with OXT are consistent with a role for OXT in adult human social behavior (Guastella and Macleod, 2012). In mice in which *Oxt* has been constitutively deleted, adults exhibit poor social recognition behavior (Ferguson et al., 2000; Macbeth et al., 2009). Similarly, adult OXT receptor (*Oxtr*) knock-out mice also display evidence of poor social recognition behavior (Takayanagi et al., 2005; Lee et al., 2008a; Macbeth et al., 2009). OXTR signaling seems to play more of role in intrastrain social recognition, which is a more difficult perceptual and memory task than interstrain social recognition (Macbeth et al., 2009). In addition, the adult *Oxtr* KO mouse is seizure-prone and has poor reversal learning, a laboratory proxy of poor cognitive flexibility (Sala et al., 2011). Maternal care is partially impaired in both lines of mice (Takayanagi et al., 2005; Pedersen et al., 2006).

Hypotheses of the underlying mechanisms that generate these behavioral phenotypes in mutant mice, in which target genes are deleted constitutively from all tissues, have been informed mostly by our understanding of OXTR ligand binding distribution in the adult mouse brain. For example, the septum, the hippocampus, the amygdala, and the piriform cortex have all been implicated. The possibility also exists that congenital OXTR loss throughout mouse development makes a significant contribution to reported disruption of adult behaviors, as has been shown for the serotonin (5-HT) 1a receptor (Gross et al., 2002). To selectively ascertain a role for OXTR in the adult brain, Lee et al generated a floxed *Oxtr* mouse, which has been used to delete *Oxtr* from selective brain areas in the adult by introduction of a cell type specific Cre recombinase (Lee et al., 2008a,b; Macbeth et al., 2009; Pagani et al., 2011). Despite a growing number of studies in mouse genetic models (Table 1), distribution data regarding the contribution of OXTR signaling during experience-dependent development has not been established in the mouse, but instead has been guided by developmental expression patterns obtained in rats (Shapiro and Insel, 1989; Snijdewint et al., 1989; Tribollet et al., 1989) and voles (Wang and Young, 1997), which differ from each other. Tritiated oxytocin and ^125^I-labeled selective antagonist mapping of developing rodent brains demonstrate robust receptor presence in postnatal development (Tribollet et al., 1989, 1991, 1992; Wang and Young, 1997). Some brain areas show dense ligand binding during postnatal development with very little expression in adults. While the location of the receptor binding varies with species, rats and voles have a peak of OXTR binding in some brain areas in the 2nd and 3rd postnatal weeks. The species diversity in developmental OXTR distribution raises the question of the similarities and differences in the characteristics of OXTR expression patterns in the developing mouse brain compared to other species.

**Table 1:** **Mouse models of oxytocin system function**.

**Gene**	**Mouse name**	**Description of manipulation**	**Original reference**
*Oxt*	OXT KO (OT KO); *Oxt*^*tm*1*Zuk*^	Congenital knockout on mixed 129/B6; *also available on C57Bl/6j	Nishimori et al., 1996; *Hammock and Levitt, unpublished
*Oxt*	OXT KO; *Oxt*^*tm*1*Wsy*^	Congenital knockout on mixed 129/B6	Young et al., 1996b
*Oxt*	Oxt/EGFP	EGFP labeled OXT-neurophysin pre-prohormone; backcrossed for many generations to C57BL/6	Zhang et al., 2002
*Oxtr*	OXTR KO; *Oxtr*^*tm*1.1*Knis*^	Congenital knockout on mixed 129/B6; *also available on C57Bl/6j	Takayanagi et al., 2005; *This report
*Oxtr*	*Oxtr*^*tm*1*(KOMP)Vlcg*^	Congenital knockout on C57BL/6	Velocigene; www.komp.org
*Oxtr*	*Oxtr^flox^*; *Oxtr*^*tm*1.1*Wsy*^	Floxed OXTR for use with Cre recombinase for selective excision	Lee et al., 2008a
*Oxtr*	*Oxtr-Venus*	Venus reporter in place of *Oxtr*; reporter positive mice are *Oxtr* hemizygous or KO; on mixed 129/B6	Yoshida et al., 2009
*Oxtr*	*Oxtr-EGFP*; Tg(Oxtr-EGFP)GO185Gsat	BAC transgenic with OXTR promoter driven expression of EGFP; theoretically does not interfere with native OXTR	GENSAT, 2003; Gong et al., 2003
*Oxtr*	OXTR-cre; Tg(Oxtr-cre)ON66Gsat	Cre recombinase driven by the OXTR promoter	GENSAT, 2003
*Oxtr*	tetOXTR	Tetracycline responsive OXTR promoter for selective regional and/or temporal over-expression; backcrossed to C57Bl/6j	L. J. Muglia pers. comm
*Oxtr*	OTR-LacZ	*Oxtr* promoter drives *LacZ* expression; *LacZ* allele results in lower *Oxtr* expression; on 129 × Balb/c mixed background	Gould and Zingg, 2003
*CD38*	CD38^-/-^	Congenital loss of CD38 which regulates secretion of OXT; On ICR x B6 X DBA	Kato et al., 1999; Jin et al., 2007

Because of observed species diversity in OXTR expression patterns and because OXTRs have not been mapped developmentally in mice, we questioned the potential role for *M. musculus*-specific developmental expression patterns that might better inform hypotheses of behavior in mouse genetic models (Table 1) in which OXTR signaling is altered genetically either directly, or indirectly through ligand alteration.

In this report, we qualitatively map and quantitate developmental expression of OXTR ligand binding in the postnatal C57BL/6J mouse brain. We also identify OXTR binding in the near term mouse embryo (E18.5) to identify potential OXT sites of action in the whole organism in the transition to postnatal life.

## Materials and methods

### Mice

Mice used in this study were bred in our animal facility from adult C57BL/6J mice obtained from Jackson Laboratories (Bar Harbor, ME). All procedures were performed after approval by the Institutional Animal Care and Use Committee of Vanderbilt University in accordance with state and federal guidelines. Timed pregnancies were generated, and females were checked daily for litters. The first morning appearance of a litter was noted as postnatal day 0 (P0). Pre-weaning litters were harvested on P0, P7, and P14. Individual litters each contributed to one time point only. Two litters were harvested immediately after weaning at P21, and individuals from several more litters were harvested at P35 and P60. E18.5 embryos were harvested from a timed-pregnant C57BL/6J mouse purchased from Jackson Laboratories and gestational age was confirmed by crown-rump length (20 ± 0.5 mm). *Oxtr* mice (*Oxtr*^*tm*1.1*Knis*^) were a generous gift from Larry Young (Emory University). We backcrossed these mice to C57BL/6J and confirmed achieving congenic status with strain specific markers (Speed Congenics Service, Jackson Laboratories, Bar Harbor, ME).

### Sex determination

Sex determination of mice aged P14 and older was performed by an experienced rater (Elizabeth A. D. Hammock) by anogenital distance. Embryonic mice and mice aged P0 and P7 were genotyped to determine genetic sex using established methods (Jimenez et al., 2003). The forward primer (5′-ccgctgccaaattctttgg-3′) and the reverse primer (5′-tgaagcttttggctttgag-3′) generate a 290 bp product from the *Smcy* gene on the Y chromosome, and a 330 bp product from the *Smcx* homolog on the X chromosome under the following thermal cycling conditions: 95°C for 7 min; 35 cycles of 93°C for 30 s, 58°C for 30 s, 72°C for 30 s; 72°C for 10 min.

### Receptor autoradiography

Each age for analyses of OXTR ligand binding consisted of animals from at least two separate litters. For the quantitative binding study, a total of 3 males and 3 females for each age were used. For the post-natal experiments, the autoradiography was performed 3 separate times with each age and sex represented. E18.5 whole embryos were harvested for examining tissue patterns of OXTR ligand binding. Brains (or embryos) were frozen in powdered dry ice and stored at −80°C until cryosectioning. Tissue was cut at 20 μm in 6 series and thaw mounted onto Superfrost Plus slides. Sections were stored at −80°C until used in the receptor autoradiography protocol. Receptor autoradiography was performed exactly as described previously (Hammock and Levitt, 2012) on rostro-caudal series of sections with 50pM of selective ^125^I OXTR ligand: ornithine vasotocin analog (vasotocin, d(CH_2_)_5_[Tyr(Me)^2^,Thr^4^,Orn^8^,[^125^I]Tyr^9^ NH_2_]; ([^125^I]-OVTA, NEX254, Perkin-Elmer, Inc., Boston, MA). Autoradiographic films (Kodak Biomax MR film, Carestream Health, Inc., Rochester, NY, USA) were developed after a 70 h co-exposure with ^14^C autoradiographic standards (American Radiolabeled Chemicals, St. Louis, MO, USA).

### Image analysis

After receptor autoradiography, slides were post-processed for acetylcholinesterase (Lim et al., 2004) and/or Nissl staining following standard protocols (Catania et al., 2008). Autoradiographic films and processed slides were scanned at 1200 dpi at 8-bit with a high resolution flatbed scanner (Epson Perfection V600, Suwa, Japan) and regions of interest were identified by comparison of the film image to landmarks in the post-processed slides. Image measurements were obtained in ImageJ (NIH, Bethesda, MD) from three consecutive sections with hand selected ROI and analyzed by interpolation “interp1,” Matlab 7.0.4 (TheMathworks, Natick, MA, USA) to the linear range of the ^14^C autoradiographic standard on the same film (Miller and Zahniser, 1987). Tissue background values were obtained from the dorsal striatum of each sample and subtracted from the region specific data to generate net values reflected in the graphed data. This resulted in average values of 0 μCi/g for each quantified brain area in *Oxtr* KO. For quantification, no adjustments were made to the images other than image inversion. Composite images for figures were created with the TurboReg (Thevenaz et al., 1998) plug-in for ImageJ using the rigid body alignment algorithm. For pseudocolor composites, the autoradiography images were adjusted for brightness to minimize the appearance of the film background.

## Results

**Figure 1:**
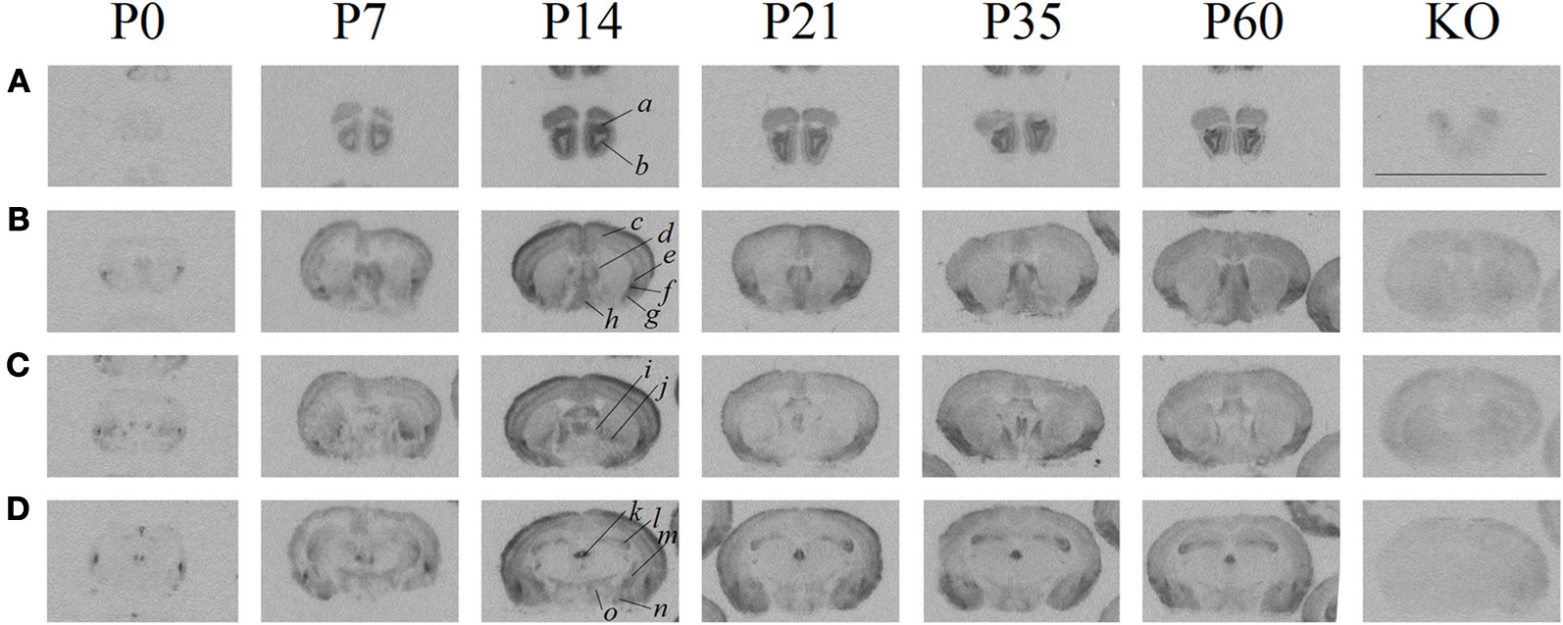
**Receptor autoradiography in C57BL/6J mice at several post-natal ages and coronal levels reveals brain areas of OXTR ligand binding, and the lack of specific OXTR ligand binding in OXTR KO brain assessed at P60.(A)** accessory *(a)* and main *(b)* olfactory bulbs **(B)** neocortex *(c)*, septum *(d)*, claustrum *(e)*, endopiriform cortex *(f)*, piriform cortex *(g)*, diagonal band of Broca *(h)*, **(C)** bed nucleus of the stria terminalis *(i)*, ventral caudatoputamen *(j)*, **(D)** periventricular thalamus *(k)*, CA3 hippocampus *(l)*, central amygdala *(m)*, medial amygdala *(n)*, hypothalamus *(o)*. Scale bar = 1 cm.

**Figure 2:**
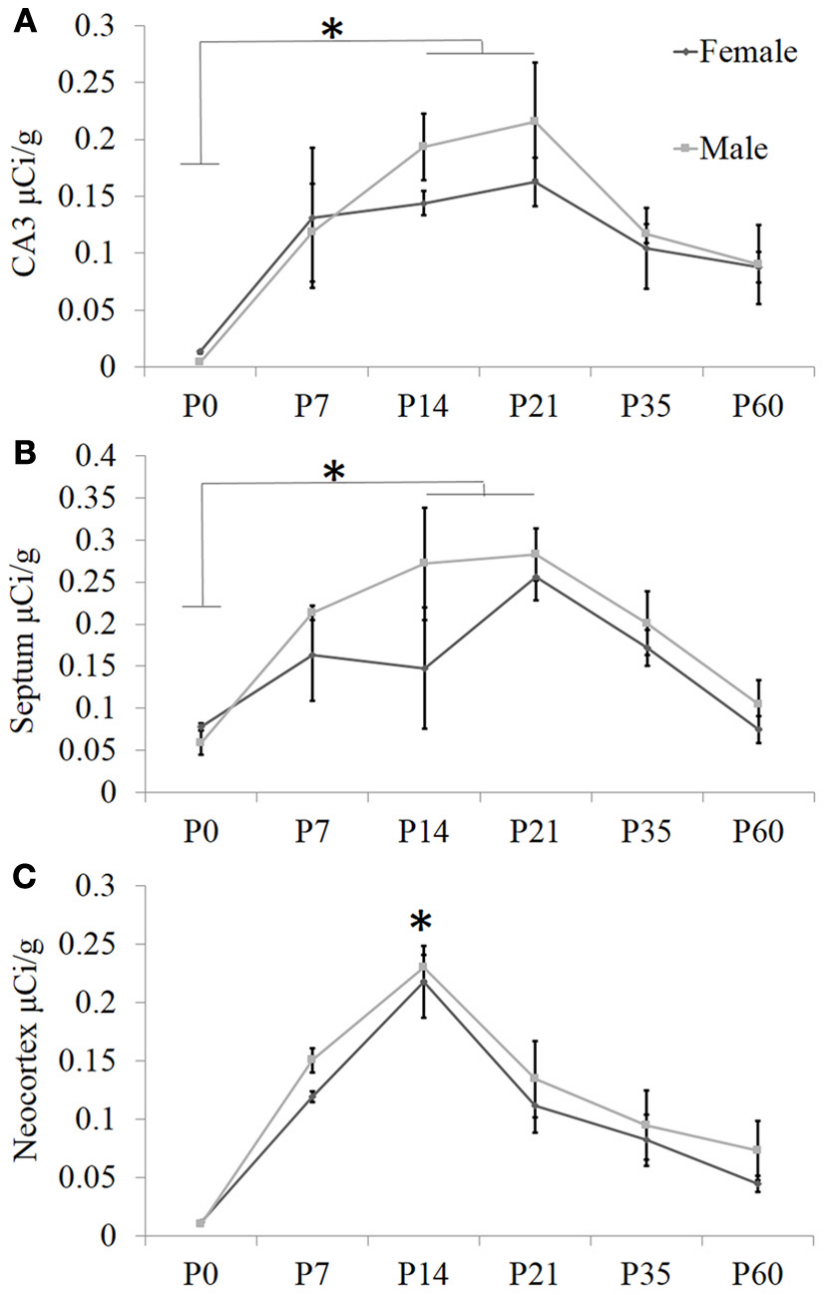
**Quantification of receptor autoradiography for OXTR in C57BL/6J mice demonstrates transient developmental profiles**. OXTR binding with highly selective OXTR ligand is evident in **(A)** the CA3 of the dorsal hippocampus, **(B)** the septum, and **(C)** the neocortex sampled at S1. For all three brain regions, there was a main effect of age. In the septum and the hippocampus, this was driven by the difference in binding between P0 and P14–P21. In the neocortex, the main effect of age was stronger and driven substantially by the peak at P14. ^*^p < 0.05.

As previously established, the ligand ([^125^I]-OVTA) used in this report is highly selective for OXTR in mice, rats, and voles as determined by displacement with a competitive unlabeled ligand (Elands et al., 1988; Insel and Shapiro, 1992; Insel et al., 1993), and by absence of specific binding in the OXTR KO mouse (Figure 1) above tissue background, as others have shown (Takayanagi et al., 2005; Lee et al., 2008a). We observed ligand binding (Figure 1) in several brain regions previously reported for high OXTR ligand binding in adult mice (Insel et al., 1993). For example, the OXTR ligand binding was evident in the lateral septum, diagonal band, piriform cortex, the central and medial amygdala, the hypothalamus, and CA3 of the hippocampus. By comparison to post-processed neuroanatomical landmarks and a neuroanatomical atlas (Paxinos and Franklin, 2001) (Allen Brain Atlas), we confirmed binding in the following areas (Figure 1) at most ages examined: accessory and main olfactory bulbs, claustrum, endopiriform cortex, bed nucleus of the stria terminalis, ventral caudatoputamen, and the periventricular thalamus. We also identified a developmental peak of ligand binding in the neocortex, evident in pre-weaning mice (Figures 1, 2).

To ascertain developmental shifts in receptor density, we quantified several brain areas that exhibited unique patterns of binding (Figure 2): the hippocampus, the lateral septum, and the neocortex. Each brain area was analyzed by Two-Way ANOVA for sex and age. The hippocampus measured dorsally at CA3 showed no main effect of sex [*F*_(1, 24)_ = 0.73, *p* = 0.40], a significant main effect of age [*F*_(5, 24)_ = 7.93, *p* < 0.001], and no sex × age interaction [*F*_(5, 24)_ = 0.41, *p* = 0.84]. The septum showed a trend for a main effect of sex [*F*_(1, 24)_ = 3.34, *p* = 0.08], a main effect of age [*F*_(5, 24)_ = 7.87, *p* < 0.001], and no sex × age interaction [*F*_(5, 24)_ = 0.75, *p* = 0.59]. OXTR density captured throughout all layers of S1 neocortex indicates no main effect of sex [*F*_(1, 24)_ = 2.31, *p* = 0.14], a main effect of age [*F*_(5, 24)_ = 26.24, *p* < 0.0001], and no sex × age interaction [*F*_(5, 24)_ = 0.19, *p* = 0.96]. Bonferroni-corrected *post-hoc* tests indicate that for the septum and the hippocampus, the P0 time-point was significantly different from the peak between P14 and P21 (*p* < 0.05). However, *post-hoc* tests for the neocortex showed that P14 was a significant peak of ligand binding which differed statistically from earlier (P0) and later (P35, P60) time points (*p* < 0.05). Thus, there are distinct regional patterns of differential OXTR ligand binding during postnatal development.

**Figure 3:**
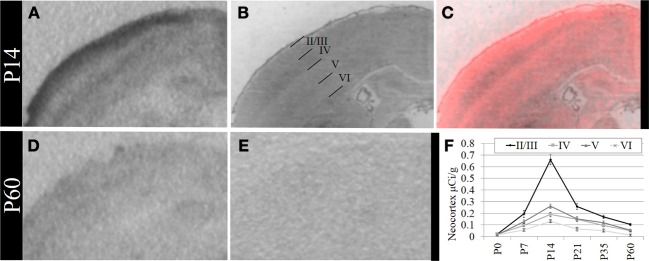
**OXTR ligand binding in the neocortex is prominent in layer II/III. (A)** OXTR is abundant in neocortical layers II/III at P14. **(B)** Nissl counterstain of section in **(A)**. **(C)** OXTR is pseudo-colored red in a composite image of panels **(A)** and **(B)**, which indicates that OXTR is prominent in layers II/III but not in layer IV or VI. OXTR is present in upper layer V. There is significantly reduced neocortical OXTR in P60 mice **(D)** which are only slightly above tissue background compared to OXTR KO P60 neocortex **(E)**. **(F)** Quantification of OXTR binding demonstrates the transient ligand binding of OXTR in upper layers across post-natal development.

Based on these data, we performed a secondary, layer-specific analysis of the developing neocortex. Post-processed neuroanatomical landmarks of the same tissue used in the receptor autoradiography facilitated localization of OXTR to layers II/III in the pre-weaning neocortex (Figure 3). We quantified OXTR binding in specific layers (II/III, IV, V, VI) across the neocortex in S1. As with the data from combined neocortical layers, there was no main effect of sex [*F*_(1, 111)_ = 0.58, *p* = 0.45], a significant main effect of age [*F*_(5, 111)_ = 141.31, *p* < 0.001], and as expected in this analysis, a significant main effect of neocortical layer [*F*_(3, 111)_ = 117.90,*p* < 0.001]. There was also a significant age × layer interaction [*F*_(15, 111)_ = 24.21,*p* < 0.001] evident in the peak binding of OXTR in layer II/III at P14. Bonferroni corrected *post-hoc* tests indicated that OXTR ligand binding density in layer II/III at P14 was significantly greater than all other ages and layers examined (*p* < 0.05).

**Figure 4:**
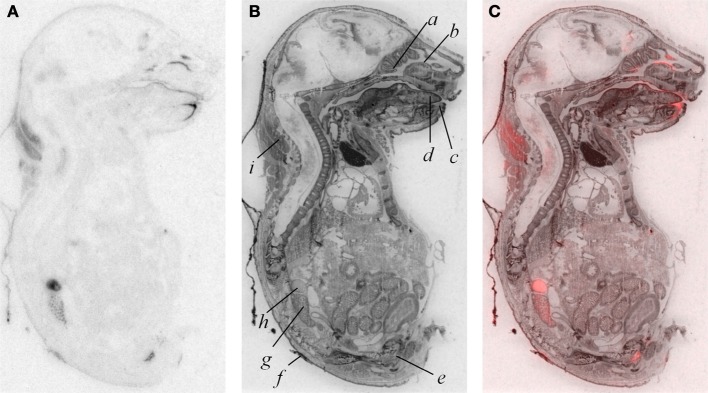
**OXTR ligand binding (A) followed by post-processing with Nissl staining (B) reveals tissue specificity of OXTR in E18.5 embryos**. Pseudocolor (OXTR in red) composite **(C)**. Olfactory turbinates/nasal epithelium *(a and b)*, mandible *(c)*, tongue *(d)*, genitourinary tract *(e)*, dermis *(f)*, kidney *(g)*, adrenal gland *(h)*, brown adipose tissue *(i)*.

In addition to modest ligand binding in the forebrain, hindbrain, and spinal cord, we observed OXTR ligand binding in several tissues throughout the E18.5 embryo (Figure 4). Dense binding was evident in a very limited number of peripheral tissues, including the dermis, brown fat, adrenal gland, kidney, genitourinary tract, testes (not shown), the olfactory sensory epithelium, and the oral cavity including the tongue, palate, and the anterior mandible.

## Discussion

The present study demonstrates an unexpected, transient developmental profile of OXTR ligand binding throughout the neocortex in the developing mouse that is in part different from other species examined previously. These data have direct implications for the interpretation of functional data from mice, as the major model of genetic perturbation of OXTR signaling. Other groups have previously mapped OXTR in developing rodents, using this ligand. Tribollet et al. (1989) and Shapiro and Insel (1989) described the postnatal development of OXTR ligand binding in the rat. They observed continuously high levels of binding in the accessory olfactory bulb, the ventral subiculum, and the central amygdala, which is similar to what we observe in mice. Unlike mice, however, rats show persistent binding in the dorsal caudate-putamen; mice do not show evidence of specific binding in this region at any age. Also unlike mice, rats exhibit transient binding in the dorsal subiculum and anteroventral thalamus. As previously described (Shapiro and Insel, 1989; Tribollet et al., 1989), rats showed dramatic transient OXTR ligand binding in the neocortex, but the expression was limited to the midline cingulate neocortex. The data in rats contrast with our findings in mice, which demonstrate potential for a broader neocortical role for OXTR outside of limbic neocortex. Wang and Young (1997) mapped OXTR with this ligand in postnatal vole brains, with a particular focus on the septum, although the images in that paper demonstrate transient (although not quantified) postnatal OXTR binding throughout the neocortex of voles as well. The unique and species-specific developmental profiles of OXTR distribution patterns allow for a potential neural substrate of species differences in OXT-dependent developmental trajectories. Perhaps as in adults (Insel and Shapiro, 1992; Young et al., 1996a; Young, 1999; Campbell et al., 2009; Ophir et al., 2012), species differences in OXTR distribution patterns in the developing brain may give rise to important species differences in maturation of social behaviors. Despite the variations in expression patterns of OXTR, rodent species in general are consistent in the timing of the transient developmental peak- neocortical OXTR peaks in the 2nd and 3rd postnatal week, paralleling the major time period of synaptic wiring and pruning in subcortical and cortical forebrain regions (Levitt, 2003; Li et al., 2010). This may provide some clues regarding developmental sensitive periods for the early role of OXTR in the ontogeny of circuits that mediate behavioral development.

In rats, Tribollet et al. (1989) defined two areas with post-pubertal onset of ligand binding: the ventromedial nucleus of the hypothalamus and the islands of Calleja of the olfactory tubercle. While we detected some ligand binding in both of these areas of mice, we were unable to detect a similar post-pubertal onset in these areas or any other related structures (data not shown). This is likely due to an actual lack of adult onset of ligand binding in mice. While unlikely, it is possible that there are technical differences in sampling through these nuclei, which would have missed limited regions of expression, due to the smaller brain size of the mouse.

Our discovery of transient OXTR throughout the developing mouse neocortex permits additional interpretations of data from Sala et al. (2011), who report that the OXTR KO mouse has lower seizure thresholds and impaired cognitive flexibility in the reversal of the T maze. The authors conclude that perhaps the loss of OXTR in the adult hippocampus is the mechanism responsible for the lower seizure thresholds. An alternative interpretation is that developmentally transient neocortical OXTR expression influences experience-dependent neocortical development that may be disrupted in the OXTR KO mouse, contributing to lowered seizure thresholds. Orphan data from the initial reports of the OXT KO mouse support this hypothesis: there was robust c-Fos activation present in S1 neocortex of the adult OXT KO mouse after exposure to a social stimulus, compared to an absence of c-Fos activation in the adult WT mouse after similar exposure (Ferguson et al., 2001). Because there are very few OXTR receptors in the adult neocortex, it is possible that this activation difference in adult OXT KO reflects atypical or labored neural processing of social information after a developmental trajectory that could not use OXT signals to shape experience-dependent neocortical development. As observed in the hippocampus (Sala et al., 2011; Owen et al., 2013) and the infralimbic medial prefrontal cortex (Ninan, 2011), OXTR may contribute to signal-to-noise processing throughout the entire neocortex by regulating excitatory and inhibitory balance during post-natal development. The hypothesis that OXT, via OXTR, shapes the experience-dependent plasticity of the entire neocortex during the onset of multisensory integration could be tested using conditional mouse lines in which gene expression would be manipulated with temporal specificity.

Given some of the developmental differences in OXTR across species, there are challenges in determining which animal models provide the most relevant translation to understand the role of OXT signaling in human brain development. Resolving this dilemma becomes especially important with the increased momentum to understand a potential therapeutic role for OXT in autism spectrum and other neurodevelopmental disorders (Bartz and Hollander, 2008; Modi and Young, 2012; Miller, 2013). While a combination of approaches are most likely to yield insight, descriptive data from BrainSpan Allen Institute gene expression data set and from Kang et al. (2011) reveal a peak of *OXTR* mRNA expression in multiple neocortical regions in postmortem tissue from neonates and infants, with lower levels prenatally and in adolescents and adults. This developmental epoch corresponds to high levels of experience-dependent plasticity for the acquisition of face discrimination (Pascalis et al., 2002, 2005) and language recognition (Kuhl, 2004), as well as peak synaptogenesis (Huttenlocher and Dabholkar, 1997). Perhaps developmental mouse models of OXT system function will be valuable for elucidating a role for OXT in mammalian experience-dependent neocortical development.

A by-product of our developmental study was the generation of unique data prenatally at E18.5, a time proximate to the transition to parturition. Parturition is an important developmental time-point with exposure to high levels of maternal OXT (Kuwabara et al., 1987; Douglas et al., 2002; Tyzio et al., 2006; Khazipov et al., 2008; Mazzuca et al., 2011). Prenatal binding of this OXTR ligand has not been examined in the whole embryo. We observed dense OXTR ligand binding in several embryonic tissues, involved in the regulation of homeostasis and are of great interest in the potential mechanisms of the transition to postnatal life.

These whole embryo OXTR binding data lead to testable novel hypotheses. One testable hypothesis would be to consider the possibility that OXTR in the developing adrenal gland contributes to the stress hyporesponsive period (SHRP)- the period of perinatal development, during which time adrenal activity is suppressed including reduced adrenal sensitivity to ACTH (Rosenfeld et al., 1991) (i.e., less corticosterone release). If OXTR contributed to the SHRP, we would expect a developmental regulation of the levels or function of OXTR in the adrenal gland, with changes in adrenal OXTR function around post-natal day 12–14.

We also observed robust OXTR ligand binding in the oral cavity in near-term embryos, which has not been described previously. In the adult mouse, OXTR has been identified on a subset of glial-like type I taste cells, and in some cells on the periphery of taste buds, where OXT can elicit calcium signals (Sinclair et al., 2010). Our embryo data suggest the following testable hypothesis: OXT present in either amniotic fluid during parturition or in breast milk (Takeda et al., 1986) could activate these receptors to initiate a cascade of signals to help orient the newborn to maternal cues.

OXTR in specific peripheral tissues of the term embryo may facilitate a physiologically coordinated transition to postnatal life. We do not yet know if these peripheral tissues expressing the receptor at E18.5 continue to express OXTR at the same levels, or if there are developmental changes in OXTR in these areas. The data observed in the postnatal brain presented here suggest that OXTR in the neocortex is well-positioned to play a role in the experience-dependent developmental plasticity of the neocortex. These are tractable hypotheses that may lead to a far more detailed understanding of the mechanisms through which OXTR signaling may orchestrate the transition to postnatal life and the developmental emergence of a species-relevant repertoire of social and/or OXT-modulated behaviors (Hammock and Levitt, 2006).

## Author contributions

Elizabeth A. D. Hammock designed and performed the ligand binding studies and data analysis, and Elizabeth A. D. Hammock and Pat Levitt prepared the manuscript.

### Conflict of interest statement

The authors declare that the research was conducted in the absence of any commercial or financial relationships that could be construed as a potential conflict of interest.
